# Risk factors associated with renal crescentic formation in pediatric Henoch–Schönlein purpura nephritis: a retrospective cohort study

**DOI:** 10.1186/s12887-020-02404-2

**Published:** 2020-11-02

**Authors:** Yong-Rui Song, Wan-Liang Guo, Mao Sheng, Qiang Lin, Xue-Ming Zhu, Xiao-Zhong Li

**Affiliations:** 1 Department of Radiology, Children’s Hospital of Soochow University, 92 Zhongnan Street, Suzhou, 215025 Jiangsu China; 2 Department of Nephrology and Immunology, Children’s Hospital of Soochow University, 303 Jingde Road, Suzhou, 215003 Jiangsu China; 3 Department of Pathology, Children’s Hospital of Soochow University, 92 Zhongnan Street, Suzhou, 215025 Jiangsu China

**Keywords:** Risk factors, Crescent formation, Henoch-Schönlein purpura nephritis, HSPN

## Abstract

**Background:**

The long-term prognosis of Henoch-Schönlein purpura (HSP) depends on the severity of renal involvement, and crescent formation is considered an important risk factor for poor prognosis of Henoch-Schönlein purpura nephritis (HSPN). The objective of this study was to evaluate factors affecting crescent formation in children with HSPN.

**Methods:**

Demographic factors, clinical characteristics, and laboratory data of children with HSPN with or without crescents were retrospectively analyzed. Univariate and multivariate logistic regression analyses were used to determine the risk factors of crescent formation in HSPN.

**Results:**

A total of 191 children with HSPN were enrolled in the study. There were 107 (56%) males and 84 (44%) females, with a median age of 7 years (range: 2 years–15 years). International Study of Kidney Disease in Children (ISKDC) grading was used to divide subjects into two groups: those without glomerular crescent formation (ISKDC grades I–II, *n* = 146 cases) and those with glomerular crescent formation (ISKDC grades III–V, *n* = 45 cases). Logistic regression analysis showed that higher urinary white blood cell (WBC) count (*OR* = 3.300; 95% CI, 1.119–9.739; *P* = 0.0306) and higher urinary microalbumin/creatinine ratio (ACR) (*OR* = 25.053; 95% CI, 1.354–463.708; *P* = 0.0305) were independent risk factors for the formation of crescents in HSPN. The area under the receiver operating characteristic curve of urinary WBC and ACR were 0.753 and 0.698 respectively, with the Hosmer and Lemeshow goodness-of-fit test (*P* = 0.0669, *P* > 0.05).

**Conclusion:**

These results suggest that higher urinary WBC count and ACR should be strictly monitored for children with HSPN. Adequate clinical intervention for these risk factors may limit or prevent renal crescent formation.

**Supplementary Information:**

The online version contains supplementary material available at 10.1186/s12887-020-02404-2.

## Background

Henoch-Schönlein purpura (HSP), also known as IgA vasculitis, is the most common systemic vasculitis in children [1], with an incidence of 20 cases per 100,000 children annually [2]. HSP is considered a self-limiting disease; however, the long-term prognosis depends on the severity of renal involvement [3]. Renal involvement, particularly HSP nephritis (HSPN), may occur in 34% of HSP children [4], although most children with HSPN have the opportunity to recover completely. HSPN may progress to renal failure in 2 to 20% of individuals [5, 6].

As the clinical symptoms and signs of HSPN vary greatly, it is difficult to predict the outcome and severity of HSPN [7]. Renal biopsy is the gold standard for evaluating the severity and prognosis of HSPN. Certain pathological features may be of value in predicting the prognosis of HSPN [7]. The existence of crescents is a prominent histological feature of HSPN and constitutes the basis of the pathological classification promulgated by the International Study of Kidney Disease in Children (ISKDC). Renal biopsy histological grade (grades I–II vs. grades III–V) score based on crescentic ISKDC pathological classification strongly predicts the prognosis of children with HSPN [8]. Also, in children, crescentic nephritis (ISKDC grades III–V) is considered an important risk factor for poor prognosis of HSPN [9–11]. Studies have reported the risk factors of renal involvement in HSP [12, 13]. However, the discovery of risk factors for progression to crescentic HSPN (ISKDC grades III–V) is an important issue for children with HSP with known renal involvement. To the best of our knowledge, risk factors in children for progression to crescentic HSPN have not been reported.

## Methods

### Patients

This study is a single-center, retrospective study of 191 children under the care of the Department of Nephrology and Immunology of the Children’s Hospital of Soochow University from January 2016 to October 2019. A patient was diagnosed with HSP in the presence of palpable purpura with lower limb predominance (mandatory criterion), along with one of following symptoms: abdominal pain; arthralgia or arthritis; renal involvement; and IgA deposition on histopathology [14]. The diagnosis of HSPN was by clinical presence of hematuria and/or proteinuria within 6 months of the course of Henoch-Schönlein purpura [15]. Hematuria was classified as either gross hematuria or microscopic hematuria (≥ five red blood cells/high power microscopic field three times a week). Proteinuria was diagnosed based on any of the following criteria: (1) routine urine qualitative analysis showing positive urine protein three times within 1 week; (2) 24-h urine protein quantification > 150 mg or urinary protein/creatinine (mg/mg) > 0.2; or (3) a urine microalbumin above normal, three times within 1 week.

Renal biopsy and pathological examination were performed to confirm the diagnosis of HSPN and to determine the severity of renal injury. Clinical treatment was carried out according to the Kidney Disease Improving Global Outcomes (KDIGO) guidelines [15, 16]. All the patients hospitalized with HSPN were preliminarily discharged after active treatment and after their clinical symptoms and signs were under control. Next, they were followed up via outpatient services every 2 weeks or once a month depending on the level of proteinuria until their condition was under control. HSPN patients with crescent formation were treated with glucocorticoids combined with cyclophosphamide pulse therapy, once per month, routinely. The renal outcomes of patients with HSPN during hospitalization are listed in the Supplementary text.

The study protocol was approved by the Institutional Ethics Review Committee at the Children’s Hospital of Soochow University and the supervising local health ministry. Written informed consent was obtained from the legal surrogates of the subjects following a detailed description of the purpose of the study.

### Procedures

Data collection included demographic data, clinical manifestations, laboratory data and renal biopsy characterization. Demographic data included the gender, age, and weight of the patients. Information about clinical manifestations, including joint pain, abdominal pain, recurrence of skin rash and HSP, season during diagnosis, food intolerance, and history of recent upper respiratory tract infection, were collected. Blood samples were collected to test the following components: whole blood cell counts and C-reactive protein (CRP) level; standard blood and biochemistry studies for renal function indicators (such as serum uric acid, blood urea nitrogen, and serum creatinine), complement and immunoglobulin levels, lymphocyte subset assessment (such as CD3+, CD4+/CD8+), coagulation spectrum, total cholesterol and triglyceride, and anti-streptolysin O and mycoplasma immunoglobulin level determination. Additionally, urine samples were obtained for white blood (WBC) and red blood cell (RBC) counts, as well as protein and creatinine quantitation. Among urinary protein levels, in addition to the urinary microalbumin/creatinine ratio (ACR), we analyzed several sensitive indicators of early renal damage: N-acetylglucosaminidase (NAG), microglobulin and urinary transferrin (TRU) [17]. Proteinuria was measured after 24 h urine collection, and defined as protein excretion 150 mg. Also, urine samples were investigated using positive dipstick test with qualitative examination of levels of urinary occult blood and protein (− to +++). Urinary WBC and RBC counts were tested using the urine sediment count method. Additionally, stools were tested for occult blood using the colloidal gold immunological method to monitor gastrointestinal involvement. This study had quantitative and qualitative variable data for 52 possible indicators.

All renal biopsy specimens were examined by light microscopy and immunofluorescence. Specimens were read by two pathologists (a chief physician and a deputy chief physician) who remained blind to clinical history and laboratory data. Two physicians, the chief in the department of pediatric nephrology and immunology, graded the pathological results. The biopsy results were graded according to the classification of the ISKDC [18]: (I) minimal alterations; (II) mesangial proliferation; (IIIa) focal or (IIIb) diffuse proliferation or sclerosis with < 50% crescents; (IVa) focal or (IVb) diffuse mesangial proliferation or sclerosis with 50–75% crescents; (Va) focal or (Vb) diffuse mesangial proliferation or sclerosis with > 75% crescents; and (VI) membranoproliferative-like lesions.

### Statistical analysis

Univariate statistical analyses were performed using Statistics Analysis System (SAS) software version 9.2 (SAS Institute Inc., Cary, NC, USA). Variable data were grouped according to two histopathological categories: those without glomerular crescent formation (ISKDC grades I–II) and those with glomerular crescent formation (ISKDC grades III–V). The quantitative data were analyzed using *t*-Test (homogeneity of variance) or Wilcoxon rank-sum test (heterogeneity of variance). The chi-square or Fisher’s exact test (dichotomous data) and the Wilcoxon two-sample test (multi-categorical data) were carried out on the qualitative data. Stepwise logistic regression analysis, performed on SAS 9.2, was used for multivariate analysis to assess the risk factors for crescentic HSPN. The odds ratios (OR) and the 95% confidence intervals (CI) were calculated. The diagnostic efficacy of the logistic regression model was evaluated by the receiver operating characteristic curve (ROC) and the Hosmer and Lemeshow goodness-of-fit test. The confidence interval was 95%, and for all tests the level of statistical significance was defined as 0.05.

## Results

A total of 191 children with HSPN received their first renal biopsy as part of this study, which included 107 (56%) males and 84 females (44%), with a median age of 7 years (range: 2–15 years). According to ISKDC criteria, subjects were divided into two groups: those without glomerular crescent formation (ISKDC grades I–II, *n* = 146 cases) and those with glomerular crescent formation (ISKDC grades III–V, *n* = 45 cases). All patients in the crescent group were ISKDC grade IIIa–IIIb, which accounted for 23.56% of the total number of cases. There were no individuals classified as ISKDC grades IV–VI.

Among the 52 variables assessed by univariate analysis, there were significant differences in 18 variables between the two groups. The variables in the qualitative data set were abdominal pain, repeated rash more than three times at diagnosis, urinary occult blood, and urinary protein levels (Table 1). The variables in quantitative data were C-reactive protein, WBC, platelet, complement component 3, total cholesterol, triglyceride levels, 24-h urinary microalbumin, 24-h urinary total protein, urinary RBC count, urinary WBC count, N-acetylglucosaminidase (NAG), α1-microglobulin, urinary transferrin (TRU), and urinary ACR (Table 2).

**Table 1:** Univariate analysis of the factors associated with qualitative data in HSPN patients with and without crescent formation

Variable	Grade I-II *n* = 146	Grade III-V *n* = 45	X^2^	P
Gender, Male/Female	79/67	28/17	0.9033	0.3491
Age, ≥8/< 8 years	87/59	28/17	0.0414	0.8389
Age, ≥10/< 10 years	50/96	17/28	0.0786	0.7792
Food intolerance, Severe/<Severe	51/80	16/24	0.0147	0.9035
Upper respiratory tract infection, Yes/No	22/118	11/34	1.7709	0.1833
Joint pain, Yes/No	60/82	20/24	0.1404	0.7079
Abdominal pain, Yes/No	46/96	25/20	7.7830	0.0053
Occult blood in stool, Yes/No	40/103	17/27	1.8058	0.1790
Recurrence of HSP, Yes/No	22/120	12/33	2.8679	0.0904
Season, Winter-Sping/Summer-Autumn	80/65	23/22	0.2282	0.6329
Recurrence of skin rash, ≥3/< 3 times	9/136	7/39	3.6940	0.0383*
Recurrence of skin rash, Yes/No	61/84	26/19	3.4140	0.0646
Level of urinary protein, −/+/++/+++	81/32/28/5	1/8/18/18	61.1805	<0.0001
Level of urinary occult blood, −/+/++/+++	14/39/58/35	1/5/10/29	21.6862	<0.0001

^*^Fisher’s exact test

**Table 2:** Univariate analysis of the factors associated with quantitative data in HSPN patients with and without crescent formation

Variables	Grade I-II *n* = 146	Grade III-V *n* = 45	P
C-reactive protein, mg/l	3.6629 ± 8.0603	6.5598 ± 11.712	0.0307^*^
White blood cell count, × 10^9^/l	10.029 ± 4.0135	11.723 ± 3.8933	0.0135
Lymphocyte, %	28.412 ± 12.924	24.107 ± 13.352	0.0540
Platelet count, ×10^9^/l	309.17 ± 88.675	343.07 ± 82.524	0.0239
Complement component 3, g/l	1.1476 ± 0.1881	1.2171 ± 0.1938	0.0330
Complement component 4, g/l	0.2555 ± 0.0857	0.2822 ± 0.0875	0.0707
Immunoglobulin A, g/l	2.2246 ± 0.8366	2.3653 ± 0.8168	0.3237
Immunoglobulin G, g/l	9.601 ± 2.495	9.1013 ± 2.3659	0.2371
Immunoglobulin M, g/l	1.181 ± 0.5038	1.2353 ± 0.5734	0.5425
Total cholesterol, mmol/l	4.2461 ± 1.2149	5.1396 ± 1.5511	<0.0001
Triglyceride, mmol/l	1.2288 ± 0.7094	1.6627 ± 0.9239	0.0006^*^
Serum uric acid, mmol/l	267.91 ± 83.918	266.55 ± 78.065	0.9232
Blood urea nitrogen, mmol/l	4.4997 ± 1.2614	4.6858 ± 1.4228	0.4031
Serum creatinine, mmol/l	39.261 ± 10.863	38.998 ± 8.1706	0.7791^*^
CD4+/CD8+, %	1.1854 ± 0.3808	1.1422 ± 0.4351	0.5220
CD3+, %	67.1528 ± 10.3439	66.1283 ± 9.3301	0.5504
CD3 + CD4+, %	32.488 ± 7.649	31.087 ± 8.6809	0.3005
CD3 + CD8+, %	28.1910 ± 6.1454	28.9500 ± 6.2194	0.4680
CD19 + CD23+, %	9.0250 ± 3.8344	8.6178 ± 3.6392	0.5300
Mycoplasma IgG, AU/l	68.3077 ± 65.7568	71.9142 ± 73.0422	0.7652
Mycoplasma IgM, COL	1.0552 ± 0.8047	1.0084 ± 0.6630	0.7329
Anti-streptolysin O, IU/ml	161.0 ± 264.4	150.0 ± 141.8	0.1712^*^
24 h urinary microalbumin, mg	269.2 ± 293.6	565.7 ± 330.4	<0.0001
24 h urinary total protein, mg	542.32 ± 821.84	1799.5 ± 1778.9	<0.0001
Urinary RBC count, /UL	94.49 ± 233.54	630.65 ± 1505.9	<0.0001^*^
Urinary WBC count, /UL	20.255 ± 63.389	80.811 ± 132.25	<0.0001^*^
N-acetylglucosaminidase, U/L	26.003 ± 16.885	34.677 ± 33.282	0.0310^*^
α1-Microglobulin, U/L	7.359 ± 4.9073	9.333 ± 5.0892	0.0204
β2-Microglobulin, U/L	0.3357 ± 0.4068	0.3231 ± 0.1824	0.5001^*^
Urinary transferrin, mg/l	24.014 ± 34.348	75.179 ± 42.057	<0.0001^*^
Urinary microalbumin/creatinine, mg/mg	0.2835 ± 0.2725	0.7933 ± 0.5126	<0.0001^*^
Prothrombin time, s	12.698 ± 1.2648	12.293 ± 1.3303	0.0659
D-dimer, μg/l	928.71 ± 1645.2	1177.3 ± 1941.1	0.4177
Fibrinogen, g/l	2.5638 ± 0.7827	2.7704 ± 0.9135	0.1394
Antithrombin III, %	110.97 ± 17.292	109.79 ± 22.1	0.7695^*^
Thrombin time, s	17.732 ± 1.72	17.704 ± 1.9175	0.9275
Activated partial thrombin time, s	32.706 ± 7.7257	30.46 ± 8.4283	0.0975
Weight, kg	32.762 ± 12.69	34.611 ± 14.598	0.4110

^*^The variance of the two samples is uneven, and the nonparametric rank sum test is used

**Fig. 1:**
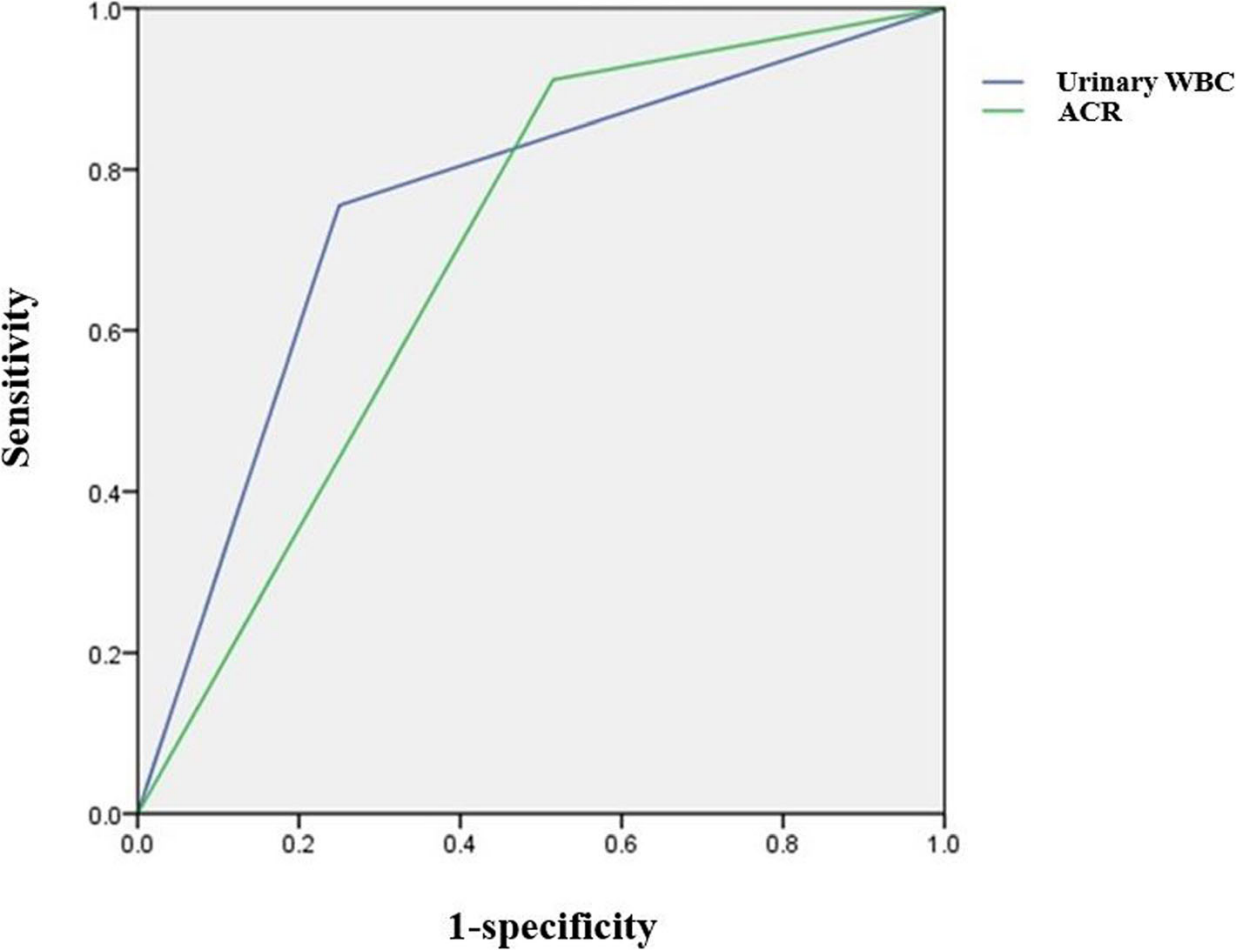
ROC curve to evaluate the diagnostic efficiency of the model. Area under the curve = 0.753 and 0.698, respectively

After multivariate logistic regression analysis, urinary WBC count (*OR* = 3.300; 95% CI; 1.119–9.739; *P* = 0.0306) and urinary ACR (*OR* = 25.053; 95% CI, 1.354–463.708; *P* = 0.0305) were found to be independent risk factors for glomerular crescentic renal damage (Table 3). The area under the ROC (AUC) of urinary WBC and ACR were 0.753 and 0.698, respectively (Fig. 1), with the Hosmer and Lemeshow goodness-of-fit test (*P* = 0.0669, *P* > 0.05).

**Table 3:** Logistic analysis of the risk factors in HSPN patients with crescent formation

Variable	OR (95% CI)	*P* value
Abdominal pain	2.119 (0.770–5.833)	0.1459
Recurrence of skin rash≥3times	2.050 (0.339–12.385)	0.4341
C-reactive protein	0.990 (0.241–4.069)	0.9890
White blood cell count	1.288 (0.423–3.922)	0.6561
Platelet count	1.153 (0.359–3.698)	0.8113
Complement component 3	13.666 (0.205–912.816)	0.2225
Total cholesterol	1.530 (0.523–4.479)	0.4377
Triglyceride	1.920 (0.567–6.501)	0.2943
24 h urinary microalbumin	> 999.999(< 0.001- > 999.999)	0.9520
24 h urinary total protein	< 0.001(< 0.001- > 999.999)	0.9374
Urinary RBC count	6.577 (0.646–66.966)	0.1116
Urinary WBC count	3.300 (1.119–9.739)	0.0306
N-acetylglucosaminidase	1.799 (0.147–21.969)	0.6456
α1-Microglobulin	1.856 (0.556–6.196)	0.3149
Urinary transferrin	1.086 (0.054–21.903)	0.9572
Level of urinary occult blood	1.749 (0.040–76.622)	0.7720
Level of urinary protein	> 999.999 (< 0.001- > 999.999)	0.9400
Urine microalbumin/creatinine	25.053 (1.354–463.708)	0.0305

## Discussion

In this retrospective study, risk factors were determined between pediatric patients with HSPN who had or did not have renal crescent formation (ISKDC grade ≥ III versus ISKDC grade < III). Multivariate logistic analysis showed that urinary WBC count and urinary ACR were independent risk factors for crescent formation. To the best of our knowledge, these risk factors have not previously been identified in relation to pediatric HSPN.

Our study has focused on the risk factors in pediatric patients with HSPN, as crescent formation has shown to be a poor prognostic factor [7–10]. However, there have been reports that found that histopathological findings also have poor predictive value for the prognosis of HSPN [19, 20]. Koskela et al. [19] reported the value of follow up renal biopsies in prognosis assessment from 26 patients with HSPN, and they concluded that follow-up biopsies provide limited additional information beyond clinical symptoms in HSPN outcome prediction. At the same time, results based on the Oxford classification revealed that complement C1–2 showed differences in diagnostic biopsies between the two groups, supporting results that active treatment should be commenced before fibrosis of crescents; our results also supporting this finding. We found that clinical manifestations, such as abdominal pain and recurrence of skin rash more than three times, were different between HSPN patients with and without crescents; however, they were not independent risk factors for crescent formation. Our results are consistent with study of Soylemezoglu et al. [20], in that clinical findings do not seem to predict the outcome of HSP nephritis in children. They concluded that the presence of crescents in the first biopsy did not seem to predict the outcome of HSPN in children.

Proteinuria denotes severe renal injury, and proteinuria at the time of symptom onset was associated with poor prognosis [8, 21]. This is consistent with our study, where multiple proteinuria indicators of renal function were associated with crescent formation, based on our univariate analysis (Tables 1 and 2). However, based on logistic regression analysis, only urinary ACR was found to be independent risk factor for predicting crescentic glomerulonephritis (ISKDC grades III–V). ACR is a sensitive indicator of glomerular damage with high specificity [22]. Significant elevation in 24-h urinary protein levels and urinary protein/creatinine ratio were found in ISKDC grades IIb, IIIa, and IIIb HSPN patients compared to ISKDC grade I and IIa subjects (the area under the ROC curve was 0.767 and 0.731, respectively), suggesting that 24-h urinary protein levels and urinary protein/creatinine ratio may predict the pathological classification of HSPN [23], supporting the view that the severity of clinical manifestations in HSPN is related to the formation of crescents [10]. Other studies employed log-term follow-up to assess renal outcome in children with HSPN. 24-h proteinuria was associated with poor prognosis [21]. Additionally, multivariate analysis showed that patients with poor prognosis had a higher urinary albumin/creatinine ratio after 17 years of follow-up [8].

Increased urinary WBCs are an indicator of renal injury. Urinary WBCs with positivity defined as more than 10 cells per high power microscopic field [24], positively correlated with complement C3 in HSPN subjects, suggesting that the complement system is involved in promoting renal injury [23]. Complement-leukocyte-dependent interactions cause glomerular damage. Indeed, data indicate that in HSP subjects, WBCs may incite renal vasculitis [25]. In children with HSP, increased peripheral blood leukocyte and neutrophil counts were risk factors for small vessel injury and kidney involvement [26]. Analysis of HSPN patient samples and renal biopsies from animals with experimental HSPN found that WBCs participated in crescent formation [7]. WBC count, CRP, and humoral immune complement C3 levels were identified risk factors for crescentic nephritis in this study. Additionally, cytokine-driven neutrophilia was associated with epithelial cell proliferation and fibrosis in Bowman’s space [7]. Nevertheless, in Jang’s study, the authors concluded that the activation of the complement system does not correlate with the clinical or pathological severity of HSPN [27]. They compared the differences in pathologic findings of 35 children and 12 adults with HSPN according to C4d positivity in groups, while our results revealed that the C3 complement of the complement system is involved in promoting renal injury. In addition, the differences between the two studies may be due to the application of different study groups, and different sample sizes.

Of further novelty, we found that abdominal pain and recurrence of skin rash more than three times were possible risk factors for the formation of HSPN crescents. Again, to the best of our knowledge, such associations have not been reported previously. Other studies noted that abdominal pain and persistent purpura (> 1 month) were risk factors for pediatric HSPN [28]; purpura relapses more than four times predicted a poor prognosis of HSPN [29]. Repeated rashes in the setting of HSPN may indicate increased inflammation/neutrophilia and vasculitis.

Hematuria is one of the initial renal manifestations of HSPN [15]. Urinary red blood cell count and level of urinary occult blood were possible risk factors in our study. The published data in this regard is mixed. For example, a meta-analysis showed that hematuria and mild proteinuria with hematuria were associated with better prognosis [11]. In contrast, patients with isolated hematuria or mild albuminuria at the onset of the disease had adverse consequences [30]. These conflicting reports may be due to different treatment schemes. For example, the current strategy for HSPN treatment is mostly based on the KDIGO guidelines [16], but some experts have pointed out that the current treatment schemes for HSPN are insufficient if only the KDIGO guidelines are followed, especially for mild types of HSPN [31].

Finally, few studies have shown that platelets are involved in the process of renal injury [32]. We found that platelet count, total cholesterol, and triglyceride levels were likely risk factors for crescent formation in pediatric HSPN. All these related indexes need to be further clarified in terms of their role in the pathogenesis and the prognosis of HSP and HSPN.

There are limitations to our study. First, the study was the from a single center and the sample size studied was small. Second, further detailed classification the pathological characteristics of the glomerular crescent (cellular, cellular-fibrous, and fibrous crescent) was not completed. This is important as it may affect the accuracy of the results. In addition, our study did not assess the risk of variables in relation to other pathologic classifications such as the Oxford classification (MEST-C) and the semi-quantitative classification (SQC), all of which are reported studies in HSPN [19]. Lastly, tissue assessment was not complemented with a control group of pediatric patients with renal disease. Thus, it is not clear if the pathologic lesions and risk factor associations are limited only to HSPN.

## Conclusions

Results presented herein find that, in pediatric HSPN, increased urinary WBCs and/or urinary ACR are risk factors for worse renal pathology. In the setting of HSPN, such laboratory findings may warrant increased treatment intervention by specialists to limit renal damage.

## Supplementary Information

**Additional file 1: Supplemental Table 1.** The matched *t*-test was performed between urinary ACR tested before biopsy and after active treatment, followed by discharge of patients from the hospital.

## Data Availability

The datasets used and/or analyzed during the current study are available from the corresponding author on reasonable request.

## Abbreviations

HSPN: Henoch-Schönlein purpura nephritis
ISKDC: International Study of Kidney Disease in Children
WBC: White blood cell
ACR: Microalbumin/creatinine ratio
HSP: Henoch-Schönlein purpura
KDIGO: Kidney Disease Improving Global Outcomes
CRP: C-reactive protein
RBC: Red blood cell
OR: Odds ratios
CI: Confidence intervals
ROC: The receiver operating characteristic curve
NAG: N-acetylglucosaminidase
TRU: Urinary transferrin
AUC: The area under the ROC
C3: Complement component 3
SQC: The semiquantitative classification
