# Supplementary material for: Risk factors associated with renal crescentic formation in pediatric Henoch–Schönlein purpura nephritis: a retrospective cohort study
Source: BMC Pediatr. 2020 Nov 2;20:501. doi: 10.1186/s12887-020-02404-2 (PMC7604931; doi:10.1186/s12887-020-02404-2)
Supplement: Supplementary file 1 — Additional file 1: Supplemental Table 1. The matched t-test was performed between urinary ACR tested before biopsy and after active treatment, followed by discharge of patients from the hospital. [file 12887_2020_2404_MOESM1_ESM.docx]

All the patients hospitalized with HSPN were preliminarily discharged after active treatment and after their clinical symptoms and signs were under control. Next, they were followed up via outpatient service every two weeks or once a month depending on the level of proteinuria until their condition was under control. HSPN patients with crescent formation were treated with glucocorticoids combined with cyclophosphamide pulse therapy, once per month, six times in total, routinely [ref 15, 16]. Urinary ACR, which is a sensitive biomarker of renal outcome in patients with HSPN, was tested during the period of hospitalization. Matched t-tests were performed between urinary ACR tested before biopsy and after active treatment. Next, the renal outcomes of patients with HSPN during hospitalization are listed below (Supplemental Table 1). After standardized and active treatment, the two groups of HSPN children with or without crescent formation, achieved significant therapeutic effects, and the matched t-tests showed significant differences.

Supplemental Table 1. The matched *t*-test was performed between urinary ACR tested before biopsy and after active treatment, followed by discharge of patients from the hospital.

| Grade | ACR (before) | ACR (after) | ACR(before)-ACR(after) | | P |
| --- | --- | --- | --- | --- | --- |
| I-II (n=137） | 0.2958±0.2734 | 0.1449±0.1744 | 0.1509±0.2383 | 1.1×10^-14^ | |
| III-V (n=45) | 0.7933±0.5126 | 0.1619±0.2505 | 0.6314±0.4874 | 4.2×10^-11^ | |

All statistical analyses were performed using R, version 3.5.3. ACR (before) indicates that urinary ACR was tested before HSPN patients underwent biopsy, and ACR (after) indicates that urinary ACR was tested after active and normative treatment. ACR (before)-ACR (after) represents the matched *t*-test differences.
